# OSARI, an Open-Source Anticipated Response Inhibition Task

**DOI:** 10.3758/s13428-021-01680-9

**Published:** 2021-11-09

**Authors:** Jason L. He, Rebecca J. Hirst, Rohan Puri, James Coxon, Winston Byblow, Mark Hinder, Patrick Skippen, Dora Matzke, Andrew Heathcote, Corey G. Wadsley, Tim Silk, Christian Hyde, Dinisha Parmar, Ernest Pedapati, Donald L. Gilbert, David A. Huddleston, Stewart Mostofsky, Inge Leunissen, Hayley J. MacDonald, Nahian S. Chowdhury, Matthew Gretton, Tess Nikitenko, Bram Zandbelt, Luke Strickland, Nicolaas A. J. Puts

**Affiliations:** 1 Department of Forensic and Neurodevelopmental Sciences, Sackler Institute for Translational Neurodevelopment, Institute of Psychiatry, Psychology, and Neuroscience, King’s College London, 16 De Crespigny Park, Camberwell, London, SE5 8AF UK; 2 The Drug research University of Tasmania Group, University of Tasmania, Hobart, Australia; 3 Trinity College School of Psychology and Institute of Neuroscience, Trinity College Dublin, Dublin, Ireland; 4 Open Science Tools (PsychoPy) lab, School of Psychology, University of Nottingham, Nottingham, UK; 5 Sensorimotor Neuroscience and Ageing Research Group, School of Psychological Sciences, College of Health and Medicine, University of Tasmania, Hobart, Australia; 6 School of Psychological Sciences and Turner Institute for Brain and Mental Health, Monash University, Melbourne, Australia; 7 Department of Exercise Sciences, Movement Neuroscience Laboratory, The University of Auckland, Auckland, New Zealand; 8 Neuroscience Research Australia, Sydney, Australia; 9 Department of Psychology, Psychological Methods, University of Amsterdam, Amsterdam, The Netherlands; 10 School of Psychology, University of Newcastle, Newcastle, Australia; 11 Cognitive Neuroscience Unit, School of Psychology, Deakin University, Melbourne, Australia; 12 Department of Pediatrics, Division of Neurology, Cincinnati Children’s Hospital Medical Center, University of Cincinnati, Cincinnati, OH USA; 13 Center for Autism and Related Disorders, Kennedy Krieger Institute, Baltimore, MD USA; 14 Center for Neurodevelopmental and Imaging Research, Kennedy Krieger Institute, Baltimore, MD USA; 15 Department of Movement Sciences, Movement Control & Neuroplasticity Research Group, Group Biomedical Sciences, KU Leuven, 3001 Heverlee, Belgium; 16 Department of Cognitive Neuroscience, Faculty of Psychology and Neuroscience, Maastricht University, 6229 ER Maastricht, The Netherlands; 17 School of Sport, Exercise and Rehabilitation Sciences, University of Birmingham, Birmingham, UK; 18 Donders Institute for Brain, Cognition & Behaviour, Radboud University, Nijmegen, The Netherlands; 19 Department of Psychiatry, Radboudumc, Nijmegen, The Netherlands; 20 Future of Work Institute, Curtin University, Perth, Australia; 21 MRC Centre for Neurodevelopmental Disorders, King’s College London, London, UK

**Keywords:** Inhibition, Behavioral Inhibition, Stopping, stop-signal task, anticipation, executive functioning, opensource

## Abstract

The stop-signal paradigm has become ubiquitous in investigations of inhibitory control. Tasks inspired by the paradigm, referred to as stop-signal tasks, require participants to make responses on go trials and to inhibit those responses when presented with a stop-signal on stop trials. Currently, the most popular version of the stop-signal task is the ‘choice-reaction’ variant, where participants make choice responses, but must inhibit those responses when presented with a stop-signal. An alternative to the choice-reaction variant of the stop-signal task is the ‘anticipated response inhibition’ task. In anticipated response inhibition tasks, participants are required to make a planned response that coincides with a predictably timed event (such as lifting a finger from a computer key to stop a filling bar at a predefined target). Anticipated response inhibition tasks have some advantages over the more traditional choice-reaction stop-signal tasks and are becoming increasingly popular. However, currently, there are no openly available versions of the anticipated response inhibition task, limiting potential uptake. Here, we present an open-source, free, and ready-to-use version of the anticipated response inhibition task, which we refer to as the OSARI (the Open-Source Anticipated Response Inhibition) task.

The ability to inhibit action (i.e., behavioral inhibition) is one of the most commonly investigated topics in psychology and cognitive neuroscience. The capacity to enact behavioral inhibition varies across the lifespan and has been identified to be affected in a wide variety of clinical cohorts (Lijffijt et al., 2005; Lipszyc & Schachar, 2010; Smith et al., 2014; Williams et al., 1999), including attention-deficit/hyperactivity disorder (Barkley, 1997) and developmental coordination disorder (He et al., 2018). Broadly, the ability to inhibit actions can be differentiated into the ability to restrain prepotent actions and the ability to cancel prepared or ongoing actions (Dambacher et al., 2014). While the former is more commonly assessed via the go/no-go paradigm (Gomez et al., 2007), the latter is most often tested using the stop-signal paradigm (Aron et al., 2007; Verbruggen & Logan, 2009).

The stop-signal paradigm was developed by Vince (1948) but was only first referred to as the ‘stop-signal task’ approximately two decades later by Lappin and Eriksen (1966). The stop-signal task was popularized by Logan and Cowan (1984), who provided the first formal account of task performance using the ‘independent horse-race model’. In 2008, a free-to-use version of the task was made available by Verbruggen et al. (2008), and perhaps as a result of making the task freely available, the number of researchers using and citing the task increased dramatically (see Verbruggen et al., 2019: Appendix A).

While several variants of the stop-signal task exist, the basic requirements are for the task to contain at least two trial types: go trials and stop trials. On go trials, participants are prompted to make an overt motor response. The means of prompting the motor response (i.e., the ‘go stimulus’) can vary, with the recommendation being that the prompt to go should be neither too simple nor too difficult (Verbruggen et al., 2019). Overly simple go stimuli may result in responses that are too fast to inhibit, whereas overly difficult stimuli may result in responses that require too much deliberation (which might make the responses too easy to inhibit). On stop trials, participants are prompted to inhibit their motor response upon the presentation of a stop signal. The stop signal is typically presented after some delay (i.e., stop signal delay [SSD]) following the go stimulus. Short SSDs increase the likelihood of a participant being able to inhibit their response, while long SSDs decrease the likelihood. The modality of the stop signal can vary (Van Der Schoot et al., 2005), with the only requirement being that the signal is sufficiently salient to be perceived swiftly and accurately by participants (Verbruggen et al., 2019).

Performance on stop-signal tasks can be assessed through analysis of both overt and covert outcome measures. While stop-signal tasks provide the typical overt outcome measures common to most paradigms in cognitive neuroscience (e.g., reaction times [RTs] and accuracy for each trial that requires a response), the stop-signal paradigm is unique in that it also has the capacity to produce estimates of the covert latency of an individual’s stopping process, referred to as their stop signal reaction time (SSRT). SSRTs are estimated through the independent horse race model, which suggests that the success or failure of inhibiting an action can be conceptualized as a race between two processes: the go process (triggered by go stimuli) and the stop process (triggered by stop signals). If the stop process is able to ‘outrun’ and finish before the go process, the prepared action is canceled. Alternatively, if the stop process is unable to outrun and finish before the go process, then the prepared action will be enacted. Using the assumptions of the independent horse race model, an individual’s SSRT can be estimated based on their RTs on go trials and their probability of stopping across a range of SSDs on stop trials (Logan & Cowan, 1984; Verbruggen & Logan, 2009).

Currently, open-access versions of the stop-signal paradigm exist, with the most popular version being ‘STOP-IT’ (Verbruggen et al., 2008). STOP-IT is a choice-reaction variant of the stop-signal task. In STOP-IT, go trials begin with a fixation cross (presented for 250 ms) followed by a go stimulus. The go stimulus presented on each trial is always one of two predetermined shapes. Participants are required to press the computer key that corresponds to the shape presented to them. For example, in the original implementation of STOP-IT, participants were required to discriminate between a square and a circle with the ‘Z’ and ‘/’ keys being mapped to each respective shape, and RTs and accuracy were recorded for each trial. In stop trials of the original STOP-IT task, participants were presented with the go stimulus followed by an auditory stop signal. While originally developed as a Windows program, the task has since been adapted to work across different operating systems (see: https://www.github.com/fredvbrug/STOP-IT for more details).

An alternative to the choice-reaction variant of the stop-signal task is the anticipated-response inhibition (ARI) task. The ARI task was originally developed by Slater-Hammel in 1960 and was later implemented by Stinear and Byblow to assess the neurophysiology of focal hand dystonia (Stinear & Byblow, 2004). ARI tasks have become increasingly popular, especially in studies where it is used concomitantly with transcranial magnetic stimulation (TMS) (Coxon et al., 2006; Gilbert et al., 2019; Guthrie et al., 2018; He et al., 2019; MacDonald et al., 2014; MacDonald et al., 2017; MacDonald et al., 2021). Unlike choice-reaction stop-signal tasks, ARI tasks do not require participants to make choice responses on go trials, but instead require them to make an anticipated response in order to stop a moving indicator (typically a vertically filling bar) at a predefined stationary target (see Fig. 2a and b for an example). This predefined stationary target is one of the major advantages of ARI tasks, as consistency in movement preparation and initiation is required of the participant across trials. Indeed, a known problem with stop-signal tasks where a ‘fast as possible’ response is required following the presentation of a go stimulus is that participants engage in what is referred to as ‘strategic slowing’ (Verbruggen, Chambers & Logan, 2013). Strategic slowing refers to when participants purposefully slow down responses on go trials in order to more successfully inhibit their response on stop trials. Problematically, strategic slowing can lead to skewing of go RT distributions, which in turn can produce biased and invalid SSRT estimates (Verbruggen et al., 2013). In ARI tasks, strategic slowing is mitigated by restricting the possible range of RTs on go trials to be around the predefined stationary target (Leunissen et al., 2017, Dambacher et al., 2014 for a comparison of major outcome measures and reliability between the ARI and choice-reaction variants of the stop-signal task).

As with all experimental paradigms, the selection and implementation of a specific variant of the stop-signal task will depend on the nature of the research question. For example, in circumstances where variability of RTs and/or the frequency of omission errors are of interest, the choice-reaction stop-signal task may be preferable. Alternatively, in circumstances where there is a reason to limit the variability of a participant’s go responses, such as in TMS studies assessing the time-course of corticospinal excitability leading up to a go response, the ARI task may be preferable. Here, we developed an open-source ARI variant of the stop-signal task, with the belief that making the task open access will encourage users of the stop-signal task to also conduct experiments using ARI, which ultimately could advance understanding of inhibitory control at behavioral and neural levels. Below, we provide details and instructions for the application and analysis of this **O**pen-**S**ource **ARI** task, which we refer to as OSARI. OSARI is a free-to-use, cross-platform task programmed in PsychoPy (Peirce, 2007; Peirce et al., 2019).

## Open-Source Anticipated Response Inhibition Task

### Installation

OSARI was created using PsychoPy (Peirce, 2007; Peirce, 2019.) v2020, an open-source Python library for creating behavioral experiments. Note that an active part of package maintenance will be to ensure compatibility with more recent PsychoPy releases, and if users experience issues, we recommend users to log issues on the project GitHub page. To run OSARI, PsychoPy must first be installed (psychopy.org; see Peirce et al., 2019 for instructions). OSARI can be downloaded at the Open-source Task and Analyses Packages team’s GitHub: www.github.com/teamOSTAP/OSARI. Once the folder for OSARI is downloaded, the script ‘OSARI.py’ can be run in PsychoPy’s coder view or any python development environment with PsychoPy installed. An installation-free version of OSARI is currently in development. This installation-free version of OSARI can be run in-browser and is both mobile phone and tablet friendly. The beta is currently available at https://run.pavlovia.org/lpxrh6/osari_online/.

### Application

**Fig. 1:**
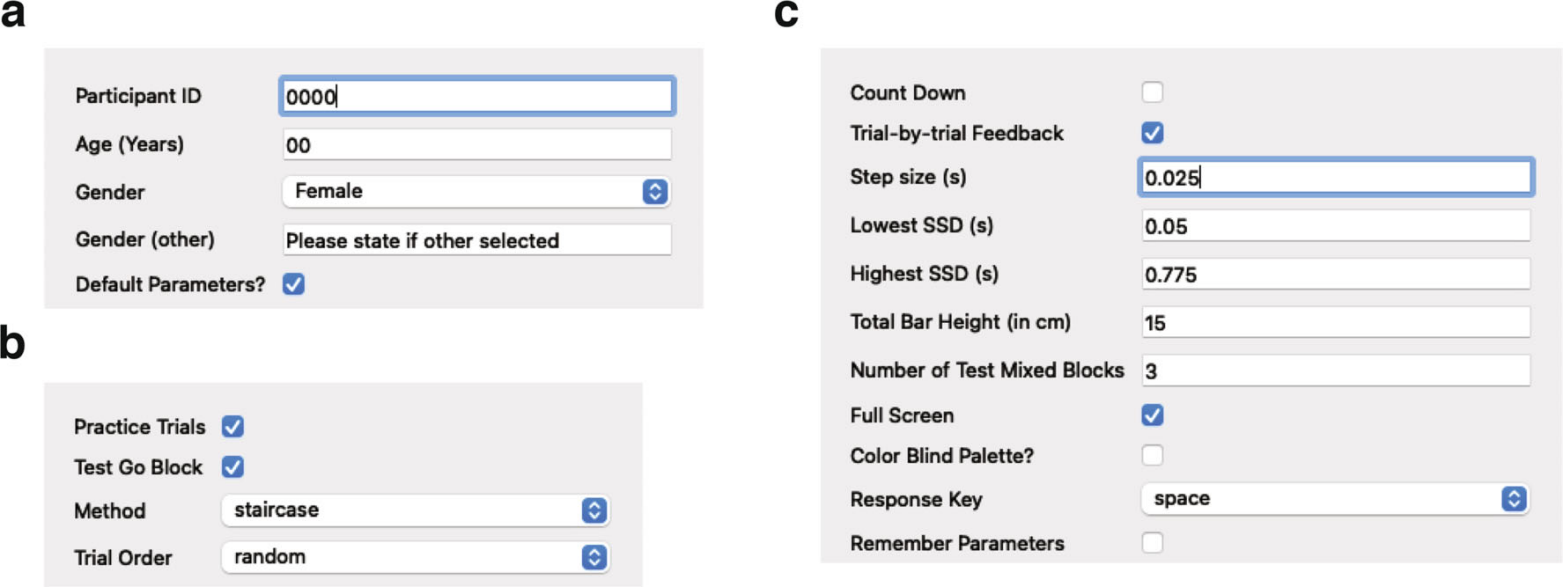
Experiment start-up dialog boxes. **a** The first ‘Participant Information’ dialog box prompts the user for basic details about the participant. The ‘Default Parameters?’ option is ticked by default and if users wish to use non-default parameters, they can untick this option. If unticked, users will be presented with two additional dialog boxes. **b** The ‘Trial Structure and Parameters’ dialog box provides the option of running the task with or without ‘Practice Trials’ and the ‘Test Go block’. Users will also be able to specify whether they want the SSDs presented using the ‘staircase’ method or using a ‘fixed’ order, as well as whether the trial order should be ‘random’ or ‘sequential’. **c** The ‘Additional Parameters’ dialog box allow users to change even more specific details of the task. Users can also select to run the task with color blind friendly colors (see: http://www.cookbook-r.com/Graphs/Colors_(ggplot2)/#a-colorblind-friendly-palette).

Once the script is running, users will be presented with a ‘Participant Information’ dialog box (Fig. 1a), which is used to collect basic demographic information about the participant. The demographic information collected in the ‘Participant Information’ dialog box can be edited through the ‘demographics.xlsx document’ by adding or removing rows. In the Participant Information dialog box, ‘Default Parameters?’ is automatically ticked. Here, if the user does not wish to use the default parameters, they can untick ‘Default Parameters?’ and two additional dialog boxes will appear. The first additional dialog box to appear is the ‘Trial Structure and Parameters’ dialog box (see Fig. 1b). The Trial Structure and Parameters dialog box gives users the option to run the task without ‘Practice Trials’ (ticked by default) and ‘Test Go Block’ (also ticked by default). Running the task without practice trials may be useful if this is not the participant’s first time completing the task. Running the task with the test go block can be helpful for assessing proactive inhibition (see discussion for details). The next dialog box to appear is the 'Additional Parameters' dialog box, which further allows users to adjust certain parameters of OSARI (e.g., the change in SSD following correct and incorrect stop; see Fig. 1c). If users wish to keep the changes they made to the default parameters, they can tick ‘Remember Parameters’ and their changes will be saved a pickle file (see: https://docs.python.org/3/library/pickle.html), which will be loaded automatically in their next run of the task.

### Block and trial structure

The default block and trial structure for OSARI are informed by Verbruggen et al. (2019)’s consensus guide to good practices for research with the stop-signal task. By default, participants will complete a practice block of go trials, followed by a test block of 30 go trials (see the ‘Proactive Inhibition’ subheading under the ‘General Considerations’ section of the discussion for an explanation of the purpose of the ‘test go block’). Once participants have completed the practice and test blocks of go trials, they will then be presented with the instructions for the stop trials, before completing a practice block of go and stop trials. By default, 10 practice go trials are presented in the ‘practice go block’ and 20 practice go and stop trials (15 go and 5 stop trials) are randomly presented in the ‘practice go and stop block’. Performance in the practice blocks will be recorded but will not be carried over to the test blocks that follow. For instance, SSD, which, in the context of OSARI, refers to the time into a trial at which the filling bar stops (e.g., if the bar stops 500 ms into the trial, then the SSD is 500 ms), resets at the start of the test block (but not between each test block).

Once participants have completed the ‘practice go and stop block’, they then complete three test ‘test go and stop blocks’. Each ‘test go and stop block’ contains a total of 80 randomly presented trials, with 60 (75%) of those trials being go trials and 20 (25%) being stop trials. If users wish to present trials in a predefined order or alter the proportion of each trial type, they can adjust the excel spreadsheet files in the ‘conditionFiles’ folder of the task and select ‘sequential’ in the ‘Trial Structure Parameters’ dialog box at task start-up. The number of rows labelled as stop and go trials corresponds to the desired trial numbers of each.

#### Stimuli

**Fig. 2:**
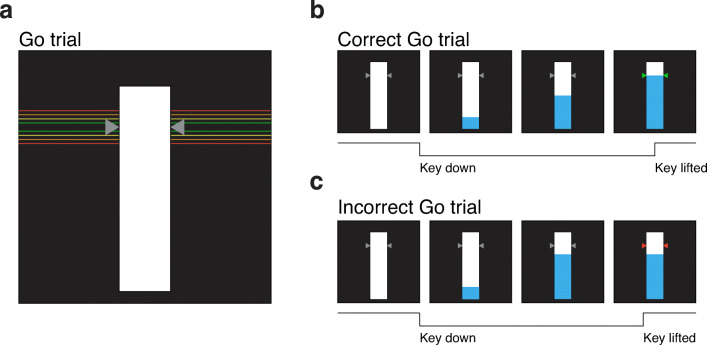
Visual Schematic of OSARI Go Trials. **a** The participant is presented with a white ‘background bar’, with two gray equilateral triangles used to depict the target. In go trials, users must depress (i.e., press and hold) a computer key to begin a trial and then release the key to stop the filling bar at the target (i.e., the innermost vertex of the two equilateral triangles). Participants are instructed to try and lift the key as close to the target as possible. On go trials, responses made within 20, 40 and 60 ms of the target will result in the triangles turning green orange and yellow. Responses made outside of 60 ms of the target will result in the triangles turning red. **b** A depiction of how a go trial unfolds. The task begins with an unfilled background bar. Once the button is pressed and held (depicted by the black line), the bar will begin to fill from the bottom up. In this example, the participant responded within 20 ms of the target line, resulting in the targets turning green. **c** In this example, the participant responded more than 60 ms below the target, resulting in the targets turning red. Note: To make the feedback, via the changing colors of the target, more accessible to a wider population, a color-blind friendly setting is available. The colors corresponding to green, yellow, orange, and red are blueish green (#009E73), yellow (#F0E442), orange (#E69F00), and vermillion (#D55E00), as these colors are unambiguous for protonopes, deuteranopes, and tritanopes (please see the excellent guide by Okabe and Ito at https://jfly.uni-koeln.de/color/).

A schematic overview of the stimuli used in OSARI is presented in Fig. 2. The default task presents participants with a 3 cm x 15 cm, vertical rectangular bar (henceforth referred to as the ‘background bar’) in the center of the screen^1^. The ‘target’ is presented as two equilateral triangles on opposite sides of the background bar. The innermost vertex of the triangles denotes where participants should try and stop the rising bar on go trials. This target appears at 80% of the total bar height. Given that the default trial length is 1000 ms, the filling bar on go trials will take 800 ms to reach the target.

At the beginning of each trial, participants are instructed to ‘Press the space key when you are ready’. The space key is used as the default response key for progressing trials and responding to targets; however, the user can change the default key using the ‘Response Key’ input in ‘Additional Parameters’ dialog box. Once the key is pressed and held, a short, randomly selected interval will occur prior to the filling of the bar. Following the variable start interval, the filling bar takes 1000 ms to fill to the top. If participants lift the key during the variable start interval (i.e., prior to the bar filling), they will receive the message: ‘Oops! You lifted too soon! Press space to restart’. If the key is lifted within the first 100 ms of the trial, participants are presented with ‘Try to stop the bar as close to the target as possible’.

**Fig. 3:**
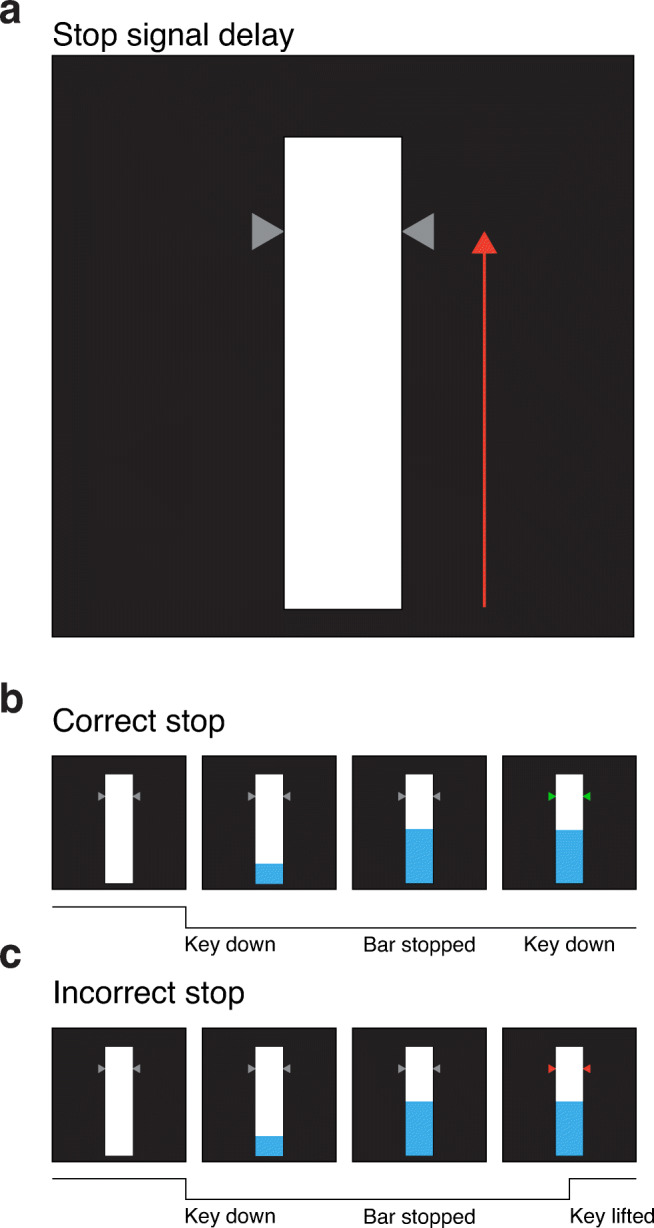
Visual schematic of OSARI stop trials. **a** SSDs are conceptualized as the time at which the bar stops, prior to reaching the target (as per the red arrow). For example, a SSD of 650 ms would mean a trial where the bar stopped 650 ms into the trial (150 ms prior to reaching the target at 800 ms). **b** An example where the bar stopped (i.e., a stop trial) prior to reaching the target location. Here the participant pressed and held the key to begin the trial, resulting in the bar filling from the bottom up. At 500 ms into the trial, the bar stopped and the participant, correctly, continued to keep the key pressed down, resulting in the targets turning green (indicating a successful stop). **c** An example of where the participant pressed and held the key to begin the trial but, incorrectly, lifted the key despite the bar stopping.

On go trials, participants are required to release a key to stop the filling bar as close to the target as possible. Feedback is given by way of the target changing color; if the filling bar is stopped, above or below the target, within 20, 40 and 60 ms, the target turns green, yellow and orange respectively. If the filling bar is stopped > 60 ms from the target line, the target turns red. (a color-blind friendly setting is available). Similarly, on stop trials, if participants successfully withhold their response following the stop signal (i.e., when the filling bar stops before reaching the target line, see Fig. 3b), the targets turn green. Note, for a stop trial, participants must withhold their response until what would have been the filling bar reaching the top of the background bar (i.e., the trial duration of 1000 ms) Alternatively, if participants do not withhold their response, the targets turn red (Fig. 3c).

#### Staircased versus fixed SSDs

OSARI allows for either staircased or fixed SSDs (using the ‘Method’ input to the ‘Trial Structure and Parameters’ dialog box). By default, SSDs are presented using the ‘staircase’ method, where the SSD starts at 500 ms into the trial and is then adjusted by participant performance. Specifically, SSDs are increased if the participant was able to accurately stop in the prior stop trial (making the theoretical probability of stopping in the subsequent trial lower), and decreased if they were not (making the theoretical probability of stopping in the subsequent trial lower). The value by which the SSD increases or decreases is determined by the ‘step size’, which by default is 25 ms, but can be adjusted to any value in the Additional Parameters dialog box. This staircase procedure aims to enable the identification of the SSD that results in a participant being able to inhibit their response only 50% of the time (i.e., P(respond|signal) = .50) and is carried across blocks. For the ‘fixed’ option, the SSD on each trial will be selected by using the corresponding value in the ‘fixedStopTime' column of the relevant condition files (i.e., ‘practiceMixedTrials.xlsx’ and ‘testBlocks.xlsx’).

#### Data output

Data files are saved regardless of whether the participant completes the task (e.g., if the task is voluntarily or involuntarily terminated), limiting unwanted data loss. The naming convention for all files is ‘ID_OSARI_yyyy_mo_d_hhmm’, where ID = participant ID, yyyy = year, mo = month in string format, d = day in numeric format, h = hour and m = minute. The unique timestamp provided to each data file enables multiple data collection sessions with the same participant ID. Two directories are generated for data output – ‘data_txt’ and ‘data’.

The ‘data_txt’ directory contains a single ‘.txt’ file compatible with the supplementary analysis script (see ‘Data analysis and visualization’ section below). The column headers in this file are as follows: ‘id’ = participant identification, ‘block’ = index of current block (per block type), ‘trialType’ = current block label, ‘trial’ = trial number within block, ‘signal’ = signal type (0 = go, 1 = stop), ‘response’ = response type (1 = go, 0 = stop), ‘ssd’ = current SSD (NaN for go trials), ‘rt’ = response time in seconds (NaN for correct stop trials). This output file is nearly identical to the output of the STOP-IT task, with the exception of the column header ‘trialType’.

The ‘data’ directory contains three files for additional information: ‘.csv’, ‘.psydat’ and ‘.log’ file. These are the default data files generated through PsychoPy’s experiment handler and are intended for the advanced user and debugging requirements. The ‘.log’ file contains chronological information on what occurred during the experiment (e.g., when keys are pressed, and stimuli are rendered). In OSARI the logging level is set to DEBUG which provides the most detailed level of logging^2^. The ‘.psydat’ file contains the saved trial handler object from the experiment that has been saved to disk. Finally, the ‘.csv’ trial provides data and trial info gathered during the task. Each row in this file corresponds to a trial. Each header corresponds to a component included on that trial. The data presented in the ‘.txt’ file can be derived from the ‘.csv’ file; however, the ‘.csv’ file provides additional information on additional parameters selected by the user (for further information on data file output from PsychoPy see https://www.psychopy.org/general/dataOutputs.html).

#### Data analysis and visualization

The .txt files contained within the ‘data_txt’ directory can be analyzed and visualized using the accompanying open-source Batch Analysis of Stop signal Task Data (BASTD) package in R (see the README file on GitHub: https://github.com/teamOSTAP/BASTD for installation guide). BASTD was developed using the R programming language (Verbruggen & Logan, 2009), version 4.0.3. BASTD has two main functions for analyzing OSARI data: ‘BASTD_analyze()’ and ‘BASTD_visualize()’. For those less familiar with R, there is a basic ‘how to’ script available (see ‘howto.R’ file). Additionally, an installation-free version of BASTD is also available as a Shiny App (see: https://bastd.shinyapps.io/shiny_bastd/).

**Fig. 4:**
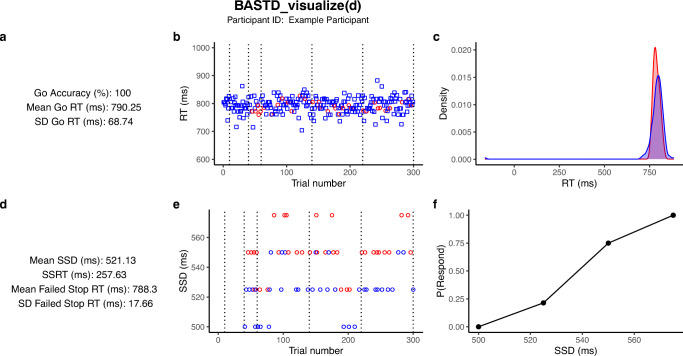
An example of the plots that are generated for each participant using BASTD_visualize(). For all plots, squares = go trials, and circles = stop trials; red = incorrect response, blue = correct response. Dotted vertical lines represent blocks. **a** Provides key descriptive data on go trials. **b** All RTs across the trials. RTs (*y*-axis) are plotted against Trial Number (*x*-axis). **c** Visualizes the density plots of the correct go and incorrect stop trial reaction times distributions. Density (*y*-axis) is plotted against RTs in ascending order (*x*-axis). **d.** Provides key descriptive data on stop trials. SSD (*y*-axis) is plotted against Trial Number (*x*-axis). **e** Depicts the change in SSD across trials. **f** Visualization of the inhibition function where the probability of responding (*y*-axis) is plotted against stop signal delay on the *x*-axis.

BASTD_analyze() analyzes the .txt output file from a single participant and requires two arguments in the function call: ‘data’ (i.e., a dataframe containing the participant’s data) and ‘task’ (i.e., a string value which can be either ‘OSARI’ or ‘STOP-IT’, depending on the data being analyzed in the data statement). BASTD_visualize() plots the data. Like BASTD_analyze(), the BASTD_visualize function only requires the same two statements: data and task. See Fig. 4 below for an example. The ‘howto.R’ file of BASTD shows users how to use these functions to batch analyze the data collected from OSARI.

## Discussion

### Summary

The use of stop-signal tasks to assess behavioral inhibition has increased dramatically over the past two decades. While many investigators have favored the use of choice-reaction variants of the stop-signal task, an increasing number of studies have adopted ARI tasks. An openly available ARI variant of the stop-signal task could encourage widespread use and could facilitate cross-study standardization. Here, we outline OSARI, allowing future investigators to easily implement and adapt the task for their desired application. Further to this, we provide an accompanying R package to batch analyze the output data saved from each participant (BASTD). With the publication of OSARI, future investigators will now have the freedom to choose the task that best suits their specific use case. Having provided the motivation for the development of OSARI, and details on how to run and analyze performance from the task, we now discuss the general and advanced considerations of task use.

### General considerations

#### ARI vs. choice-reaction stop-signal tasks

Currently, choice-reaction stop-signal tasks are being used far more prevalently than ARI tasks (see Matzke et al., n.d.; Leunissen et al., 2017). While ARI tasks (such as OSARI) and choice-reaction stop-signal tasks are based on the same underlying paradigm (i.e., the stop-signal paradigm), they may not measure the same ‘type’ of inhibition. For instance, even though Leunissen et al., 2017 reported significant associations between SSRTs estimated from performance in choice-reaction and ARI tasks, the shared variance was lower than expected (*R* = 0.48; *R*^2^ = 0.23, *p* = 0.025). The low shared variance between SSRTs estimated from performance on CR stop-signal tasks and ARI tasks could be due to several factors, including mode of response (i.e., a key press or a key release, or a simple response versus a choice response), within-individual variability of SSRT, implementation of inhibition at different points of the motor hierarchy, degree of context independence violations and/or ‘type’ of inhibition being measured.

#### Model assumptions underlying SSRT estimation

Non-parametric estimation of SSRTs is made possible through the independent horse race model (Logan et al., 2014; Matzke et al., 2018). While SSRTs can be estimated based on participant performance on OSARI (using the functions of BASTD), it is important for users to check for violations of assumptions. One prominent assumption is that of context independence, which assumes that the finishing time distribution of the go process is the same regardless of whether a stop-signal is presented. The severity and prevalence of violations of the assumption of context independence in stop-signal tasks has been an ongoing discussion. While some studies have found violations of context independence (Åkerfelt, Colonius & Diederich, 2001; Gulberti, Arndt & Colonius, 2014; Özyurt, Colonius & Arndt, 2003; Aron et al., 2007; Lappin & Eriksen, 1966; Logan & Cowan, 1984; Vince, 1948), others have not (Camalier et al., 2007; De Jong, Coles, Logan & Gratton, 1990; Hanes & Schall, 1995; Hanes & Carpenter, 1999; Osman, Kornblum & Meyer, 1986; Matzke Curley Gong & Heathcote, 2019; Coxon et al., 2006; Slater-Hammel, 1960; Stinear & Byblow, 2004; Van Der Schoot et al., 2005; Verbruggen et al., 2008; Verbruggen et al., 2019). The exact reason for this discrepancy in findings is currently unknown. A recent example of a study having identified violations of context independence includes the work by Bissett and colleagues, which highlighted that the assumption of context independence was often violated in their analysis of existing data from choice-reaction variants of the stop-signal task (Bisset et al., 2021). The violations appeared to be ubiquitous, being present across modes of responding, specific effector use or stimulus modality. In a more recent study, evidence for violations were also reported in several existing as well as novel ARI datasets, including data collected using OSARI (Matzke et al., n.d.). Importantly, within the same study, when comparing violations of context independence between data collected from a single choice-reaction stop-signal task and a variety of ARI tasks, violations of context independence appeared to be more common in ARI tasks than the choice-reaction stop-signal task. Thus, estimations of SSRT using the standard independent race model may be compromised. Given the pervasive nature of context independence violations, it is clear that we need to adjust existing models or even develop new models of inhibition that can account for these violations. We believe that a universal solution for both tasks will eventually be proposed and reach consensus. In the meantime, Matzke and colleagues have provided a solution for violations of context independence in data collected using ARI and choice-reaction (Matzke et al., n.d.). Here, a Bayesian approach was modified to estimate parametric stop-signal race models (BEESTS), accounting for ARI performance and the associated context independence violations. Using R functions implemented in the Dynamic Models of Choice R system (Heathcote et al., 2019), users can fit and check this new ‘BEESTS-CV’, and if it passes the checks, they can use it to produce valid estimates of SSRT. The code to implement BEESTS-CV can be found at osf.io/tw46u/.

#### Proactive inhibition

By default, OSARI includes a block of 30 go trials (the ‘test go block’), completed after the ‘practice go block’. The test go block provides a measurement of how participants perform on the go task before the inclusion of stop trials. A comparison of the mean and standard deviation of the go trial RTs in the ‘test go block’ and in the ‘test mixed blocks’ can be used to provide a measure of proactive inhibition (see Vink et al., 2014 for more details). While the default settings automatically include the test go block, it is possible to skip the test go block by adjusting the settings in the start-up dialog box.

#### Stop signal modality

The majority of applications of ARI tasks have used a visual rather than auditory stop signal (Coxon et al., 2007; Gilbert et al., 2019; Guthrie et al., 2018; He et al., 2018; Leunissen et al., 2017; MacDonald et al., 2017; Slater-Hammel, 1960; Stinear & Byblow, 2004; Vink et al., 2014) and OSARI is in accordance with this. As stated in the consensus paper as per Recommendation 2 (Verbruggen et al., 2019), stop signals must be obvious and salient. One of the complications introduced with the use of an auditory stop signal is that the perceived loudness of the stop signal will differ depending on the background noise, the system volume, and differences in user hardware. Indeed, the salience of the stop signal likely affects SSRT, thus users should consider this before changing the modality of the stop signal. Secondly, different software and hardware configurations can affect the latency of auditory stimuli (Bridges et al., 2020).

#### Modes of responding and recording responses

The default response method of OSARI is a button *release* rather than a button *press*. The reasoning behind this is twofold. First, a button release measures an earlier movement component of an action, whereas a button press measures the end point of an action. For estimations of RTs (and hence SSRT), it is more accurate to index the start rather than end of an action, since it removes the latency between action initiation and action termination. Second, given that the visual stimulus is a vertically filling bar, performing a vertical button release is congruent from a visuospatial perspective (though it could be argued that it feels more natural to press down to stop a vertically filling bar). For a more comprehensive comparison and discussion of RTs between button release and button press in ARI tasks, see the work by Leunissen and colleagues (Leunissen et al., 2017). If users require help using non-standard response methods and hardware, please contact us at https://www.github.com/teamOSTAP/OSARI/issues or opensourceTAP@gmail.com.

#### SSD step sizes

OSARI’s default step size (25 ms) is based on the idea that step sizes should ideally be of a size that allows the stop trials to sample across the necessary range of SSDs to determine the inhibition function (i.e., the probability of responding, given the stop signal, measured against increasing SSD) and SSRT for most participants. To illustrate the importance of this, consider a step size that is too large (e.g., 500 ms), this may only give us stop trials where a participant’s probability of responding is zero (i.e., no response) or one (i.e., response made), preventing estimation of the entire inhibition function and thus SSRT. Conversely, if the step size is too small, then the number of stop trials required to reach the SSD where a participant’s probability of responding is 50% (via staircasing) may be too high. A maximum and minimum SSD has also been implemented in OSARI, with the maximum SSD being 775 ms into the trial and the minimum SSD being 50 ms into the trial. The maximum value is to prevent SSDs being presented after where an action should have occurred (i.e., at 800 ms) and the minimum value is to present some portion of the filling bar, both of which are highly unlikely to be reached.

### Advanced considerations

#### Stimulus presentation

Although OSARI implements several *static* stimuli (the target depicted by the two equilateral triangles, instruction screens, white background bar etc.), *dynamic* stimuli are inherent to this task, with the most important stimulus being the vertically filling bar. There are at least two approaches that can be considered for rendering dynamic stimuli and for the purposes of our descriptions below, we term these approaches *‘space’* and *‘time’* approaches. In OSARI, we implement the *time* method, although we outline both here for the interested reader.

In the *space* approach for rendering dynamic stimuli, the total distance a stimulus would have to travel, or ‘fill’, within a given duration must be determined prior to the stimulus presentation. Based on the default OSARI parameters, the total distance the filling bar has to travel is 15 cm in 1000 ms (i.e., the length of the trial). From this we can calculate the distance the stimulus must travel in each frame. If we are using a 60 Hz monitor, the total distance our filling bar must travel per frame is:

$$ \mathrm{Distance}\ \mathrm{per}\ \mathrm{frame}=\mathrm{Total}\ \mathrm{distance}\ \mathrm{to}\ \mathrm{travel}\ast \left(\mathrm{Time}\ \mathrm{per}\ \mathrm{frame}\kern3pt /\kern3pt \mathrm{Total}\ \mathrm{seconds}\ \mathrm{to}\ \mathrm{travel}\right) $$

From this, the total distance our filling bar would travel per screen refresh is 0.25 cm 15 * [(1/60) / 1]. The *space* approach is dependent on consistent monitor refresh rates, which can be problematic if frame rate is not reliable. For example, if a large number of background processes are running, frames can be ‘dropped’. In OSARI, frame dropping may also result in the filling bar never actually achieving its full ‘end of trial’ height, since the filling bar would not be rendered on the dropped frame. The *time* approach offers one approach to avoiding this issue.

In the *time* approach, the total time it should take for a stimulus to move the required distance (i.e., the bar to fill) is first determined. Using OSARIs default parameters, the total time it should take for the filling bar to fill from bottom to top is 1000 ms. In the *time* approach, the start time of each trial is recorded as well as the time at the start of each subsequent frame with the time elapsed since the start of the trial used to determine where the top of the filling bar should be drawn. More specifically, the position of filling bar would be calculated as:

$$ \mathrm{Position}=\mathrm{Total}\ \mathrm{distance}\ \mathrm{to}\ \mathrm{travel}\ast \kern3pt \left(\mathrm{Time}\ \mathrm{elapsed}\ \mathrm{in}\ \mathrm{seconds}/\mathrm{Trial}\ \mathrm{duration}\ \mathrm{in}\ \mathrm{seconds}\right) $$

Thus, if the elapsed time at the start of a particular frame is 0.337 s, the height of the filling bar will be 5.055 cm [15 * (0.337 / 1)]. The benefit of this approach is that it is more resilient to unreliable frame rates (i.e., dropped frames), since time elapsed since the start of the trial is queried at the start of each frame.

Given that the *time* approach was conceptualized to be more robust, this method was selected for rendering the filling bar in OSARI. Still, we recommend users make an effort to limit the likelihood of frame drops where possible. An easy way for users to reduce the likelihood of frame dropping is by limiting the number of background programs running. Users can also assess the reliability of frame duration prior to testing (in PsychoPy v2020.1.2 this can be implemented through using the demos > timing > timeByFrames.py). For the most accurate measure of timing, users should measure timing of stimulus presentation with their own experimental set up using a photodiode (for a thorough outline of timing measurement for stimulus presentation and response times see Bridges et al., 2020).

### Future directions

OSARI provides a first-place framework to move forward in developing understanding of anticipatory response inhibition using ARI tasks. We welcome community development via GitHub^3^. For example, since PsychoPy provides easy methods for interfacing with external hardware and for taking studies online, OSARI has clear scope to be extended for use with neuroimaging and brain stimulation paradigms, as well as for use online. Future releases may also facilitate additional task parameters, for example, a bimanual version of OSARI to assess selective inhibition (see Coxon et al., 2007, for an example). The numerous directions for growth illustrate how the OSARI project will continue to make ongoing contributions to the study of inhibitory control as well as encourage transparent, open-science practice within our research community.

## Footnotes

1 Because we specify the height of these objects in centimeters, users must ensure that the size details in the ‘Monitor Center’ of PsychoPy are accurate to their current set-up to ensure accurate size presentation across screens.

2 We particularly recommend users take note of any WARNING messages in their log file, which provides information such as possible inconsistencies between the requested monitor size and detected monitor size which would affect the size of stimuli (e.g., ‘WARNING: User requested fullscreen with size [1440, 900], but screen is actually [1920, 1200]. Using actual size’).

3 Log issues and make pull requests for bug fixes and feature integration at: https://github.com/teamOSTAP/OSARI/pulls
